# The Role of Fibers in the Femoral Attachment of the Anterior Cruciate Ligament in Resisting Tibial Displacement

**DOI:** 10.1016/j.arthro.2014.08.033

**Published:** 2015-03

**Authors:** Yasuyuki Kawaguchi, Eiji Kondo, Ryo Takeda, Keiichi Akita, Kazunori Yasuda, Andrew A. Amis

**Affiliations:** a Department of Mechanical Engineering, Imperial College London, London, England; b Musculoskeletal Surgery Group, Imperial College London School of Medicine, Charing Cross Hospital, London, England; c Department of Sports Medicine and Joint Surgery, Hokkaido University Graduate School of Medicine, Sapporo, Hokkaido, Japan; d Unit of Clinical Anatomy, Graduate School, Tokyo Medical and Dental University, Tokyo, Japan

## Abstract

**Purpose:**

The purpose was to clarify the load-bearing functions of the fibers of the femoral anterior cruciate ligament (ACL) attachment in resisting tibial anterior drawer and rotation.

**Methods:**

A sequential cutting study was performed on 8 fresh-frozen human knees. The femoral attachment of the ACL was divided into a central area that had dense fibers inserting directly into the femur and anterior and posterior fan-like extension areas. The ACL fibers were cut sequentially from the bone: the posterior fan-like area in 2 stages, the central dense area in 4 stages, and then the anterior fan-like area in 2 stages. Each knee was mounted in a robotic joint testing system that applied tibial anteroposterior 6-mm translations and 10° or 15° of internal rotation at 0° to 90° of flexion. The reduction of restraining force or moment was measured after each cut.

**Results:**

The central area resisted 82% to 90% of the anterior drawer force; the anterior fan-like area, 2% to 3%; and the posterior fan-like area, 11% to 15%. Among the 4 central areas, most load was carried close to the roof of the intercondylar notch: the anteromedial bundle resisted 66% to 84% of the force and the posterolateral bundle resisted 16% to 9% from 0° to 90° of flexion. There was no clear pattern for tibial internal rotation, with the load shared among the posterodistal and central areas near extension and mostly the central areas in flexion.

**Conclusions:**

Under the experimental conditions described, 66% to 84% of the resistance to tibial anterior drawer arose from the ACL fibers at the central-proximal area of the femoral attachment, corresponding to the anteromedial bundle; the fan-like extension fibers contributed very little. This work did not support moving a single-bundle ACL graft to the side wall of the notch or attempting to cover the whole attachment area if the intention was to mimic how the natural ACL resists tibial displacements.

**Clinical Relevance:**

There is ongoing debate about how best to reconstruct the ACL to restore normal knee function, including where is the best place for ACL graft tunnels. This study found that the most important area on the femur, in terms of resisting displacement of the tibia, was in the central-anterior part of the femoral ACL attachment, near the roof of the intercondylar notch. The testing protocol did not lead to data that would support using a large ACL graft tunnel that attempts to cover the whole natural femoral attachment area.

The ideal outcome for an anterior cruciate ligament (ACL) reconstruction is to restore the native knee function, both the stability and kinematics. In single-bundle ACL reconstruction, it has been accepted that the femoral ACL graft tunnel should be placed close to the roof of the femoral intercondylar notch. However, studies of the outcome of ACL reconstruction in vitro^1,2^ and in vivo^3,4^ showed that such knees had sometimes retained abnormal instability. Rotational deficits were related to placing the ACL graft close to the roof of the femoral intercondylar notch.^5,6^ Recently, therefore, techniques in which the ACL graft attachment is spread further across the anatomic attachment area have been used. These include anatomic double-bundle reconstruction of the anteromedial (AM) and posterolateral (PL) bundles^7^ and “anatomic” (i.e., placed centrally in the ACL femoral attachment) single-bundle reconstruction procedures.^8,9^

It is implicit in these stages of evolution of ACL reconstruction that the exact site of the femoral attachment of the graft is most important. Noting this, researchers have recently performed anatomic and histologic work to understand the femoral ACL attachment in more depth. Early studies divided the ACL into AM and PL fiber bundles,^10^ and their attachment areas are associated with ridges on the surface of the femur.^11^ Mochizuki et al.^12^ reported that the femoral attachment of the ACL has a dense, direct attachment of the ACL midsubstance fibers, as well as thin, membranous attachments of the fibers that spread out on the posterior condyle, termed “fan-like extension fibers.” Hara et al.^13^ confirmed that the dense midsubstance fibers attach to a narrow oval area on the lateral condyle. Iwahashi et al.^14^ described that the direct insertion of the ACL was located in the depression between the resident's ridge and the articular cartilage margin on the lateral femoral condyle, whereas Sasaki et al.^15^ reported that it was located at the anterior narrow part of the whole ACL insertion. Mochizuki et al.^16^ reported that the posterior fan-like extension area remained adherent to the surface of the femur and was not aligned with the load when the knee was flexed. This observation suggested that these fibers may have a limited role.

There have been many studies of ACL reconstruction. Some created femoral tunnels in the direct attachment of the ACL midsubstance,^7,17^ whereas others have recommended tunnels that include as much as possible of the attachment area, including the fan-like extension fibers.^11,18^ This difference may be caused by a deficit in our knowledge on how the load carried by the ACL is transmitted to the femoral attachment. There have been studies in which the ACL was separated into 2 fiber bundles^19^ or 3 fiber bundles,^20^ but those did not use recent anatomic knowledge of the attachment morphology. Simply stated, we do not know which parts of the ACL, across the femoral attachment, are most important in resisting tibial displacements. If this were known, it would—for the first time—provide objective mechanical data to guide the femoral tunnel position in ACL reconstruction procedures if the objectives of the operation include the aim to try to reproduce the load transmission behavior of the natural ACL. To obtain more detailed knowledge, the ACL should be split into many small fiber bundles.^13^

The purpose of this study was to clarify the load-bearing functions of the fibers of the femoral ACL attachment in resisting tibial anterior drawer and rotation. It was hypothesized that most of the load transmitted by the ACL would not be in the attachment of the fan-like extension fibers but would be in the central relatively narrow area, where the dense midsubstance fibers of the ACL insert directly.

## Methods

### Specimen Preparation

Eight fresh-frozen cadaveric right knees without evidence of previous injury or surgery (mean age, 76.5 years; range, 67 to 91 years) were obtained with consent and permission from the London Riverside research ethics committee. The knees were stored at −20°C and thawed before use. Each knee was prepared on 1 day and kept overnight in a refrigerator, and the biomechanical experiment was completed the following day. The tibia and femur were cut approximately 15 cm from the joint line. The fibular head was transfixed to the tibia with 2 screws to maintain its anatomic position. The skin, musculature, patella, and central part of the posterior capsule were then removed so that the tibial and femoral shafts were exposed, leaving the knee ligaments intact. The tibia and femur were placed in 60-mm-diameter cylindrical steel pots and fixed with bone cement and screws. During the preparation and testing procedure, specimens were kept moist with wet tissue paper and water spray.

### Partition of Femoral Attachment of ACL

Two transverse holes were created across the distal femur, avoiding the collateral ligament attachments and other stabilizing structures. The medial femoral condyle was separated with a reciprocating saw, starting between the cruciate ligaments and cutting proximally in the sagittal plane for 60 mm and then medially.^21^

**Fig 1:**
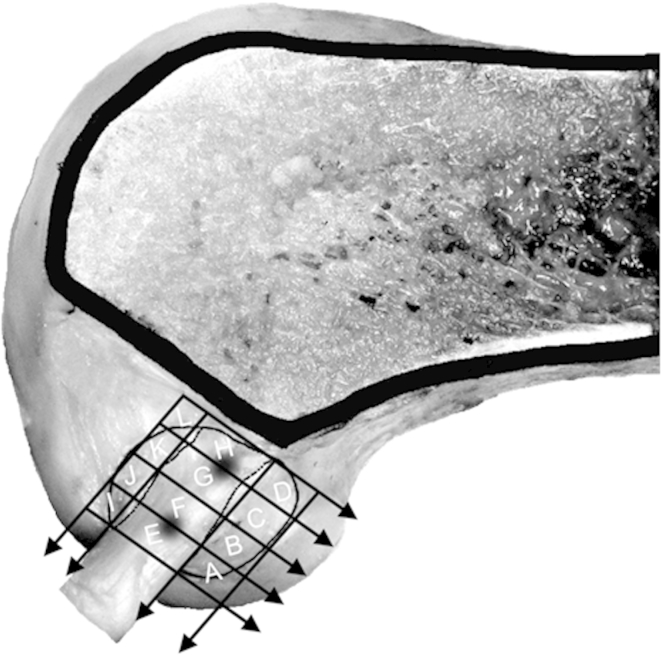
Partition of femoral ACL attachment on lateral wall of intercondylar notch. The outer lines are tangent to the ACL attachment and are oriented parallel either to Blumensaat's line or to a line joining the centers of the 2 fiber bundles of the ACL (anteromedial and posterolateral). Areas A, B, C, and D comprise the posterior fan-like extension; areas E, F, G, and H comprise the central direct attachment area; and areas I, J, K, and L comprise the anterior fan-like extension.

The morphology and dimensions of the femoral ACL attachment were assessed on the lateral intercondylar surface. The outline of the attachment with the fan-like extensions was marked (Fig 1), on the basis of visual observation of the extent of the ACL fiber attachment area. The narrow, central, direct attachment area of the ACL midsubstance was identified by flexing and extending the knee with the ACL tensed. The directly attaching fibers remained tight when the knee was flexed and extended, passing straight to the bone attachment. The posterior fan-like extension fibers were tight when the knee was near extension but remained adherent to the surface of the femur when the knee flexed, causing a distinct folding of the ACL fibers at the boundary between the posterior fan-like extension and the central direct attachment areas, as illustrated in prior anatomic studies.^12,16^ A similar process identified the boundary between the central direct fiber attachment area and the anterior fan-like extension area, in a deep fold between the bulk of the ACL and the wall of the intercondylar notch. These boundary lines were marked and were found to be straight and parallel, so another line could be drawn midway between them, along the central axis of the direct attachment area. Two more lines were drawn parallel to the central line, tangent to the outer edges of the attachment. Five parallel lines were drawn parallel to Blumensaat's line: 2 were tangent to the outer edges of ACL attachment, and then the other 3 were equally spaced between them so that the length of the central direct fiber attachment area was divided into 4 equal parts. For the geometry and position of these divisions, we used a ruler and sharp-pointed dividers, so the dimensions of the sub-areas were known within ±0.5 mm. This method divided the femoral attachment of the ACL into 12 partitions—4 central, 4 anterior, and 4 posterior—so that the femoral attachment of the ACL had been partitioned in a manner akin to a 12-square checkerboard (Fig 1). The lines defining these areas had ink rubbed into them for easy identification during the fiber-cutting surgical procedure.

**Fig 2:**
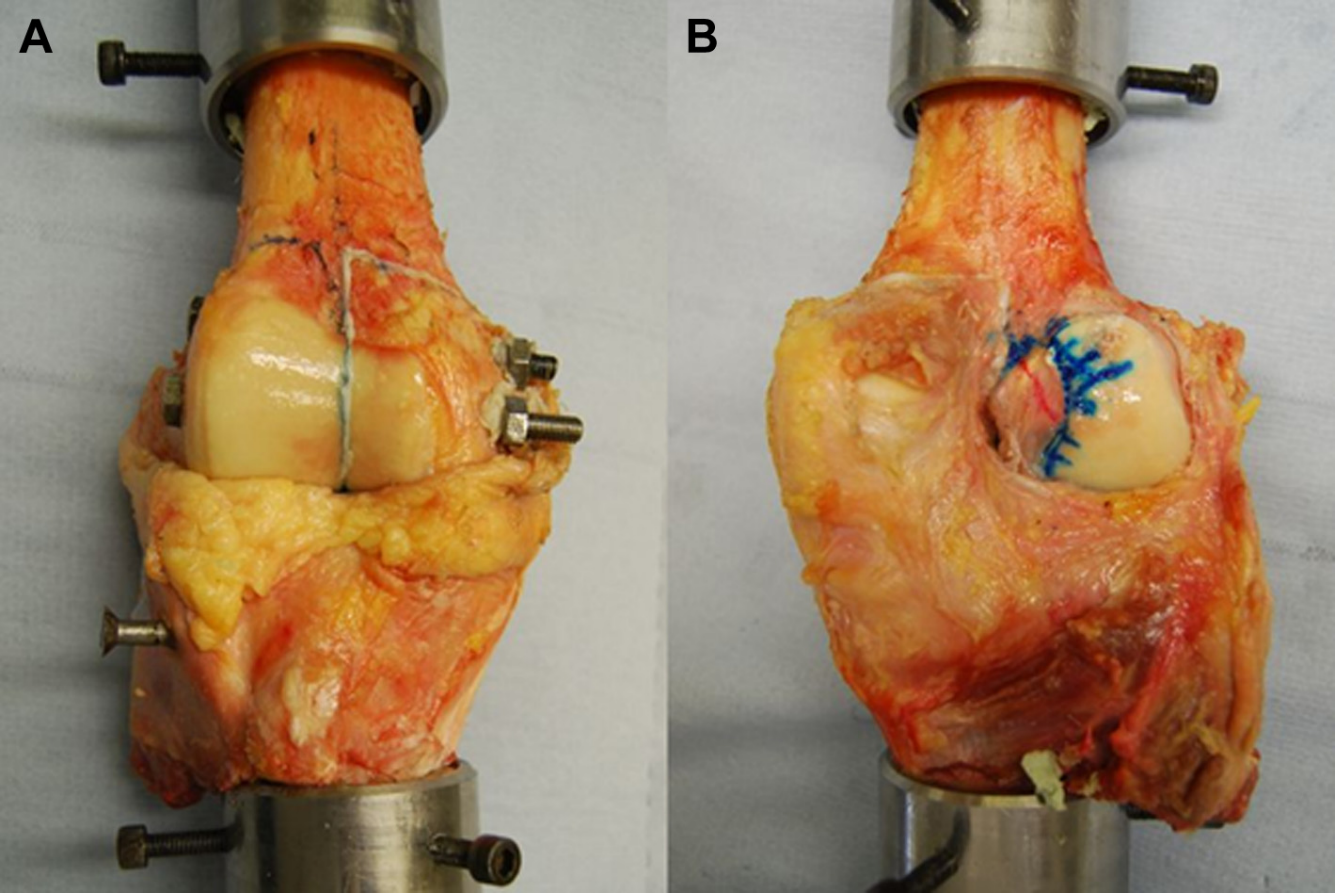
(A) The separated femoral condyle was relocated using threaded steel rods sliding through steel tubes. The separated femoral condyle was restored to the correct position using bone cement and clamped using nuts and washers. (B) The grid showing the partition of the ACL attachment has been marked on the femoral condyle around the intact ACL, through a posterior opening of the joint capsule with the knee extended.

The separated femoral condyle was relocated using threaded steel rods and then secured using bone cement, nuts, and washers^21^ (Fig 2). This defined the “intact” knee state as the starting point for the measurements of knee stability.

### Measurements of Femoral Attachment of ACL

**Fig 3:**
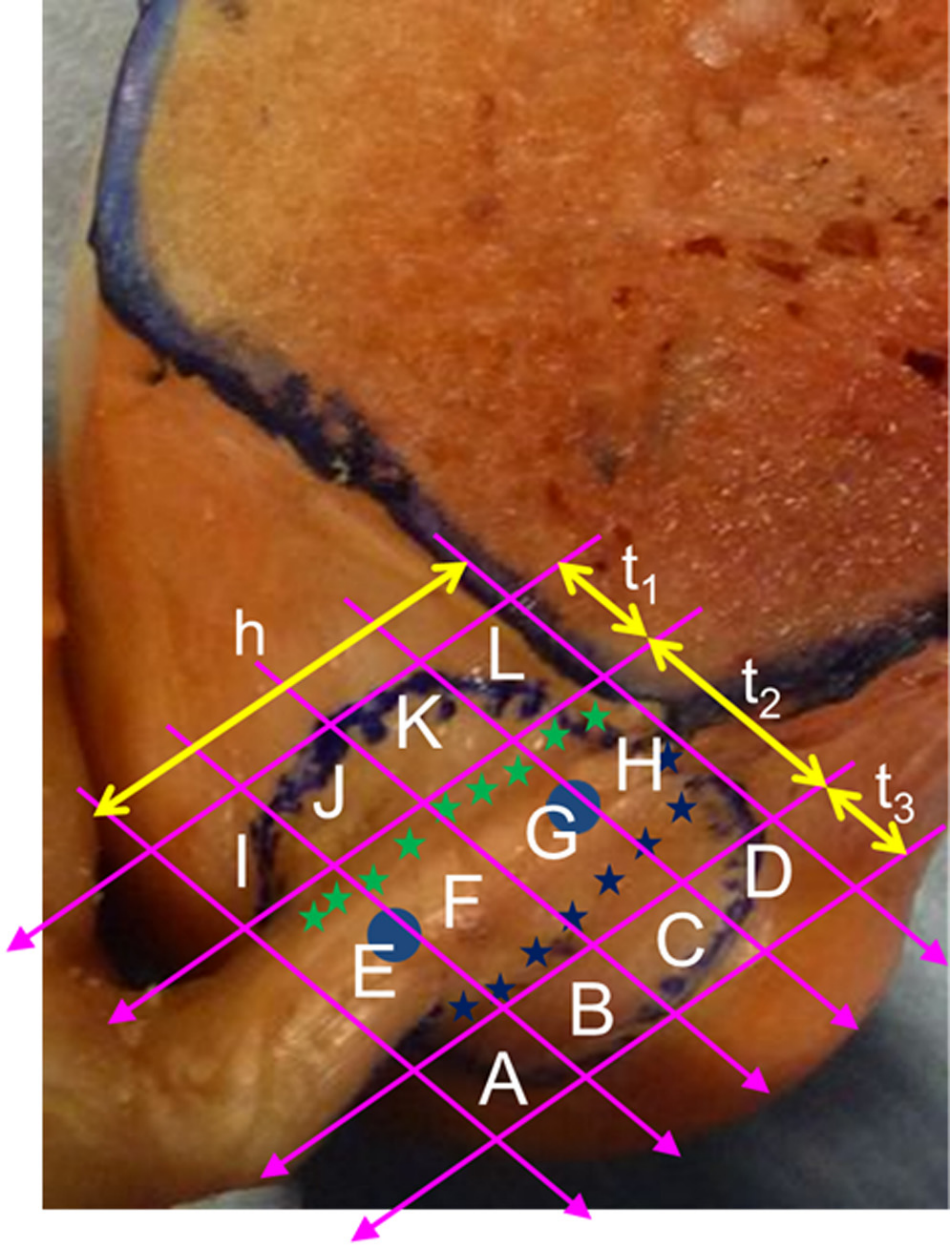
The lengths of the lines surrounding the ACL attachment were directly measured with a scale after drawing each line tangent to the edges of the ACL attachment. The blue dots show where the centers of each of the anteromedial and posterolateral bundles were judged to be; the stars show what were judged to be the boundaries between the fibers attaching directly into the bone (t_2_) and the fan-like extension areas anteriorly (t_1_) and posteriorly (t_3_). (h, length of femoral attachment.)

The ACL attachment area was measured with a scale^16^ (Fig 3) to determine the following:

• The length of the femoral attachment (h)
• The width of the attachment of the anterior fan-like extension (t_1_)
• The width of the central direct attachment (t_2_)
• The width of the posterior fan-like attachment (t_3_)

### Robotic Biomechanical Testing System

**Fig 4:**
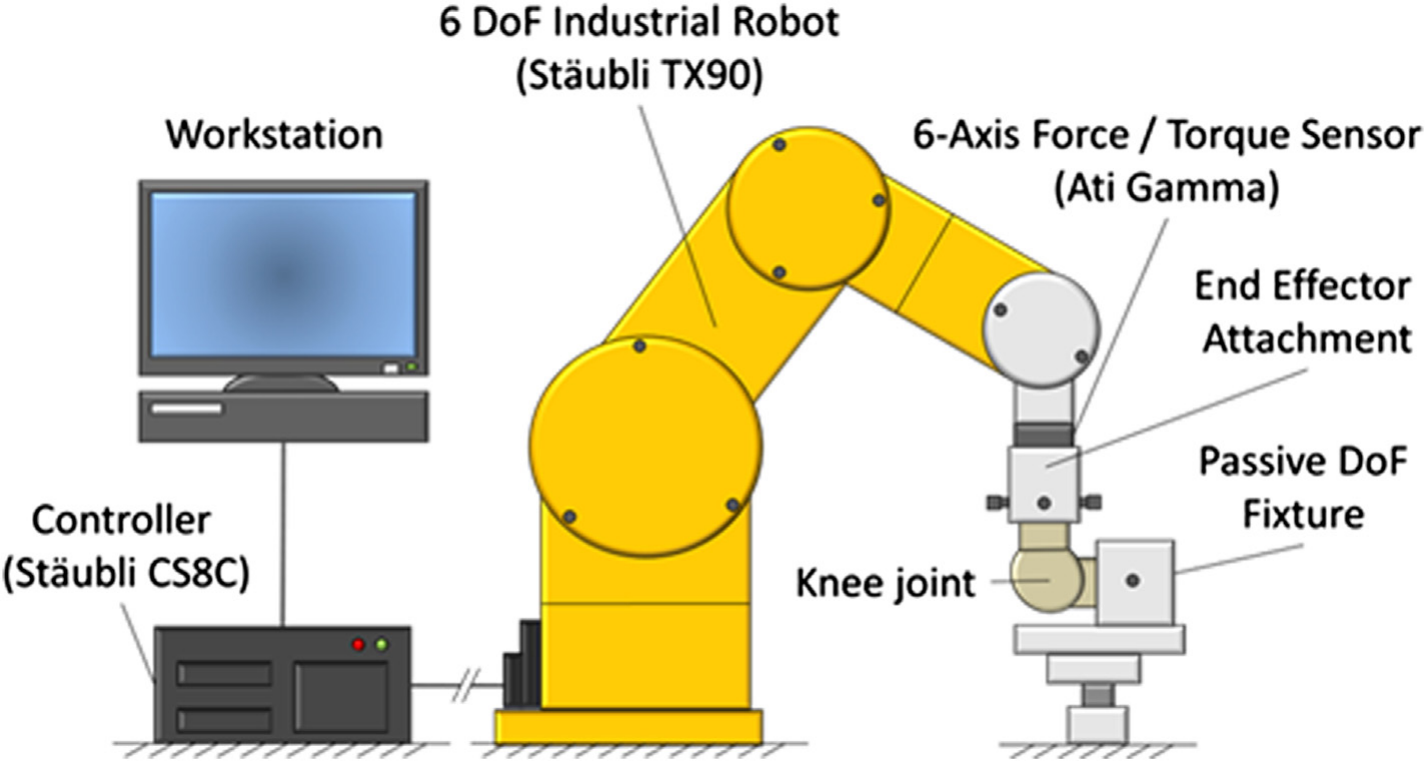
Biomechanical testing system using an industrial 6–DoF robotic manipulator and 6-axis force/torque sensor.

The anteroposterior laxity and internal-external rotation laxity were measured using a robotic knee joint biomechanical testing system. The system consisted of a 6-degrees of freedom (DoF) industrial robotic manipulator (TX90; Stäubli, Pfäffikon, Switzerland), robot controller (CS8C; Stäubli), 6-axis force/torque sensor (Gamma; ATI Industrial Automation, Apex, NC), end-effector attachment for the tibial bone pot, and fixed femoral mounting on the base of the robot (Fig 4). The robotic manipulator had a maximum load of 200 N and repeatability of 0.03 mm, whereas the force sensor range was 400 N (resolution ±0.05 N) for the z-axis and 130 N (resolution ±0.025 N) for the x- and y-axes and the torque range was 10 Nm (resolution ±0.00125 Nm) for the z-, x-, and y-axes.

### Testing Protocol

Under the active DoF release, the knee was moved from full extension (0°) to 90° of knee flexion and the positional data of the robot were recorded. During knee flexion, the force/moment sensor readings of the remaining 5 DoF were actively minimized in real time to obtain the path of passive knee motion. Afterward, the robot was operated under position control to repeat the path of passive knee motion.^22^ The robot control allowed knee laxity testing to be performed at a chosen angle of flexion. The following translations and rotations were applied to the tibia at full extension and 30°, 60°, and 90° knee flexion: (1) 6-mm anterior translation; (2) 6-mm posterior translation; (3) 10° of internal rotation at full knee extension and 15° of internal rotation at 30°, 60°, and 90° of flexion; and (4) 10° of external rotation at full extension and 15° of external rotation at 30°, 60°, and 90° of flexion. The displacing force or the torque was recorded in each situation; the robot minimized loads in the secondary DoF. Smaller displacements were used when the knee was in extension because of the higher stiffness than in flexion to reduce the possibility of causing irreversible changes.

The intact knee was tested using the aforementioned protocol; thereafter, partial cutting of the ACL at the femoral attachment was performed from a posterior approach at full knee extension. The cutting order was as follows: A + B, C + D, E, F, G, H, I + J, and K + L. By cutting sequentially down to the bone from posterior to anterior with the knee in extension (when all the fibers of the ACL were tensed in an anterodistal direction), we could be sure that the fibers associated with specific parts of the ACL attachment had been detached and that any fibers attaching anterodistal to the area that had been detached would not have been damaged; it would not have been possible to vary the cutting order with the same precision. The robotic testing was repeated in each situation. After each of the fiber bundle cuts, the reduction of displacing load indicated the resistance to tibial displacement that had been contributed by the cut fibers.

### Statistical Analysis

All data were described as mean and standard deviation. One-way analysis of variance was performed at each angle of knee flexion, with the dependent variable being the remaining force or torque applied to the specimen at the fixed tibial displacement. If a significant effect of ACL fiber cutting was detected, then specific changes were analyzed using the Tukey-Kramer post hoc test (StatView; SAS Institute, Cary, NC). The significance level was set at *P* = .05.

## Results

### Dimensions of Femoral Attachment of ACL

The length of the femoral ACL attachment (h) was 17.9 ± 2.0 mm (mean ± SD). The width of the femoral attachment of the anterior fan-like extension (t_1_) was 4.3 ± 0.9 mm, the width of the central direct attachment (t_2_) was 8.5 ± 1.1 mm, and the width of the posterior fan-like extension (t_3_) was 5.7 ± 1.6 mm.

### Restraining Actions of ACL

##### Anterior Translation

**Fig 5:**
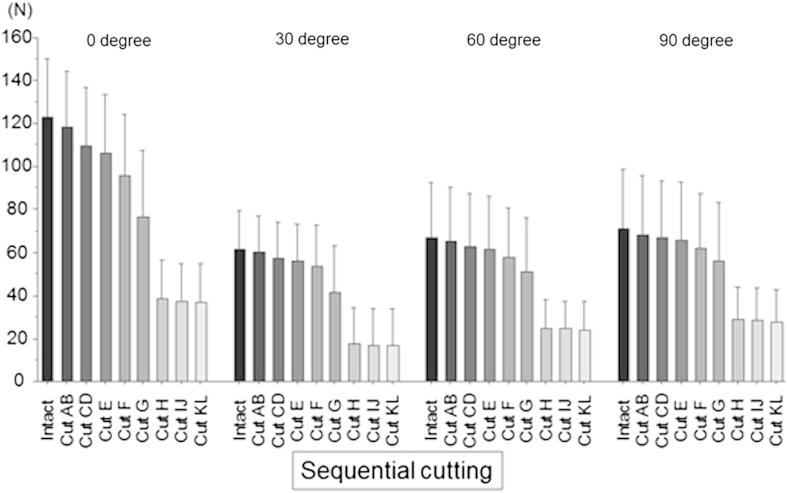
The force required to produce a 6-mm anterior translation of the tibia at 0°, 30°, 60°, and 90° of knee flexion was reduced progressively by the sequential cutting of each area in the femoral anterior cruciate ligament attachment. The cutting order was as follows: A + B, C + D, E, F, G, H, I + J, and K + L.

The force required to produce a 6-mm anterior translation of the tibia in the intact knee ranged from 61 ± 9 N at 30° of knee flexion to 123 ± 27 N at full extension. The force was reduced by the sequential cutting of the femoral ACL attachment, with *P* < .0001 at all angles of knee flexion (Fig 5).

**Fig 6:**
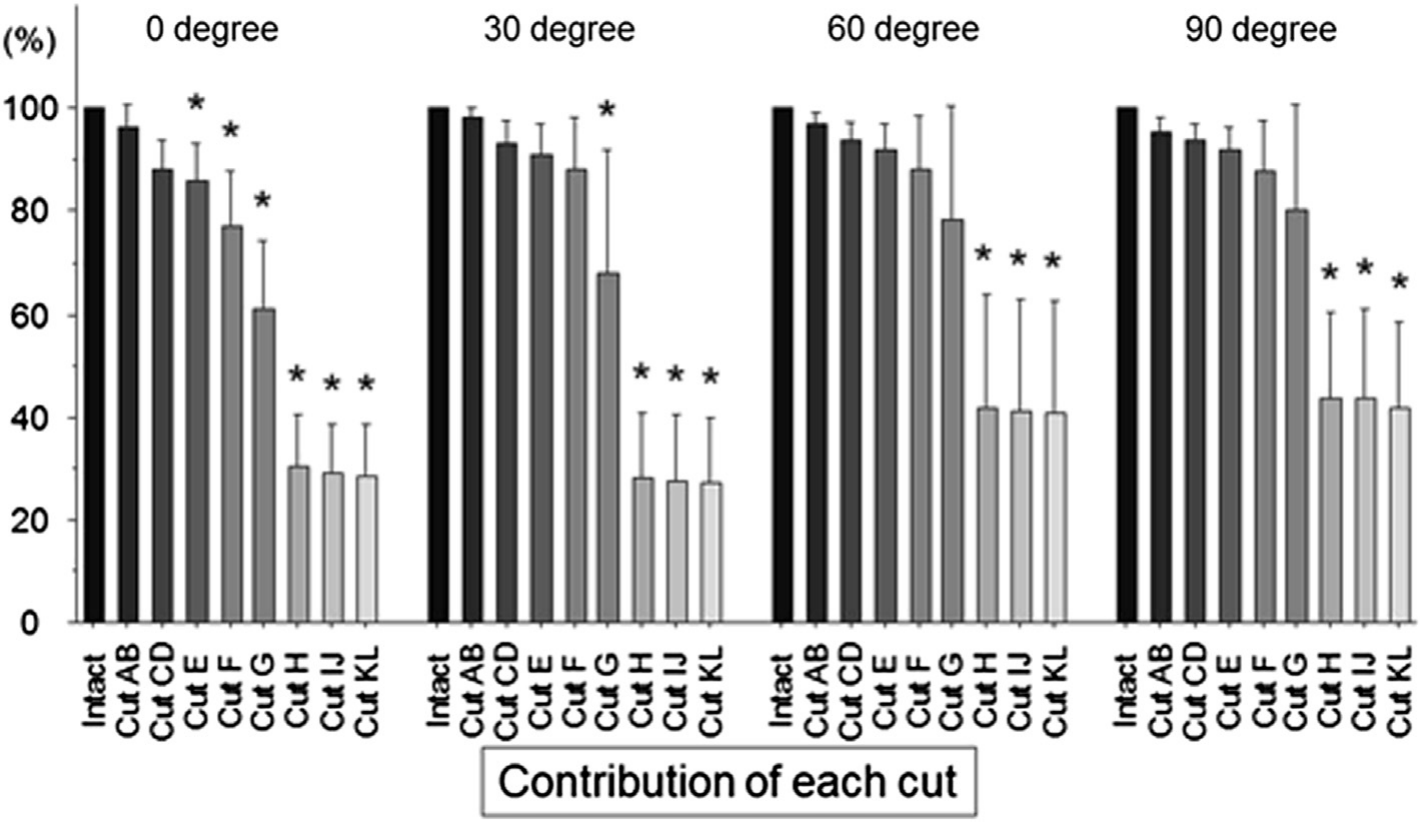
Percentage of force released by each sequential cut (i.e., “contribution of each cut”) in response to a 6-mm tibial anterior translation at 0°, 30°, 60°, and 90° of knee flexion (mean and standard deviation, N = 8). There was no significant difference after cutting areas AB and CD at any angle of knee flexion, as compared with the intact knee. Significant reductions (*P* < .05 [asterisks]) were found after cutting area E at full extension, after cutting area G at 30° of flexion, and after cutting area H at 60° and 90° of knee flexion.

The percentage of the force released by each of the sequential cuts was calculated at each angle of knee flexion (Fig 6), when the force of the intact ACL was considered 100%. There was no significant difference between the intact knee and the partially ACL-deficient knee after cutting the posterior fan-like attachment areas AB and CD at any angle of knee flexion (Fig 6). At full extension, the percentage force showed a significant decrease after cutting area E compared with the intact knee (*P* < .05). There was a significant decrease (*P* < .05) of the force after cutting area G at 30° of flexion and after cutting area H at 60° and 90° of knee flexion.

**Fig 7:**
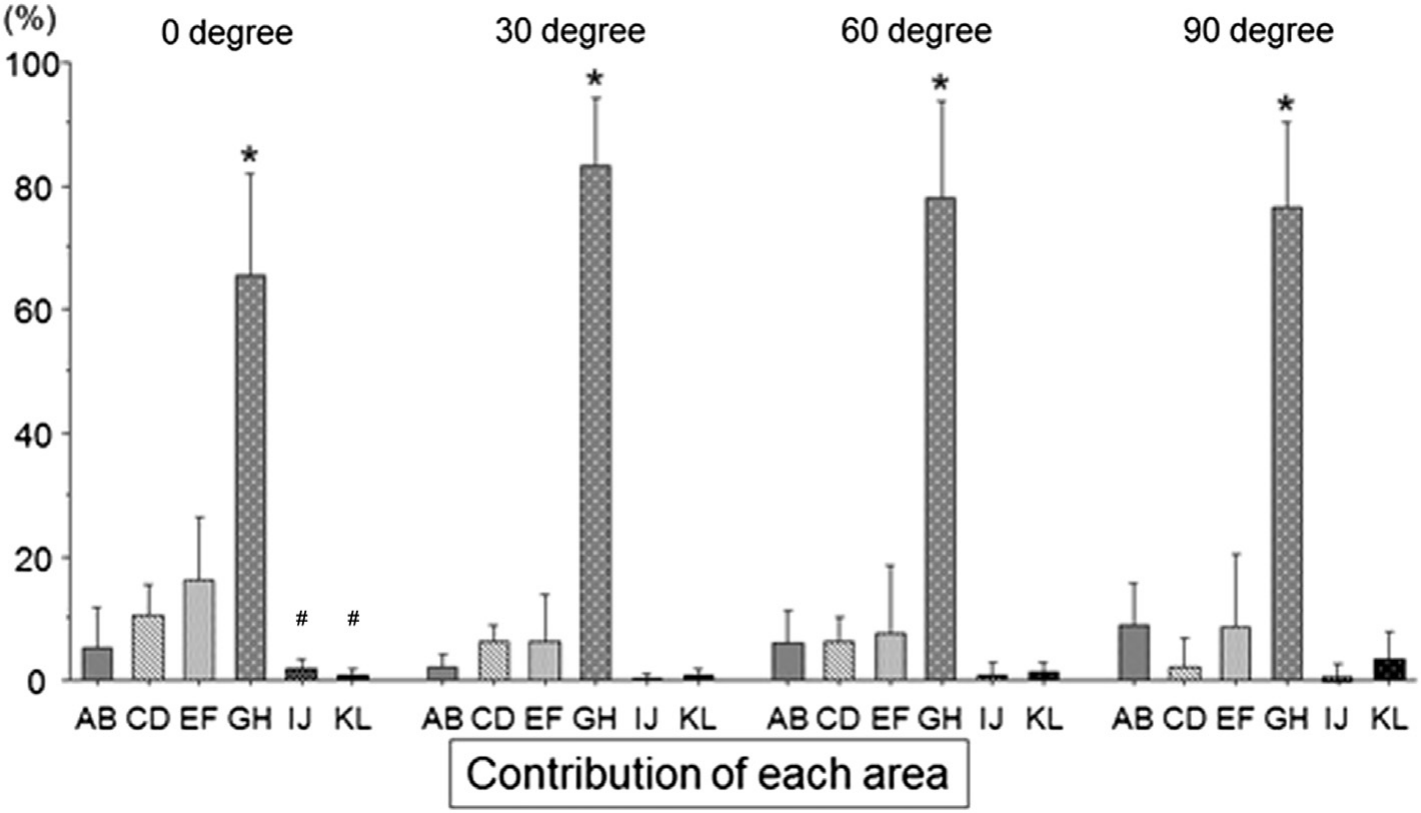
The percentage contribution of each area to a 6-mm anterior translation of the tibia was calculated, when the force of the anterior cruciate ligament in the intact knee condition was considered 100%. The percentage contribution of zones E and F and zones G and H approximately shows that of the posterolateral and anteromedial bundle attachments, respectively. The percentage contribution of zones G and H was dramatically greater at each angle of knee flexion (*P* < .05 compared with other angles [asterisks]). Pound signs indicate significant differences (*P* < .05) compared with area EF.

To analyze the tibial displacement–resisting function of the fibers attaching to each part of the femoral ACL attachment from the clinical viewpoint, the drawer force with the ACL intact was considered 100% and the percentage contributions were calculated after cutting each partition (Fig 7). Specifically, we calculated the percentage contributions of areas E + F and G + H because they approximately showed the contributions of the PL and AM bundle attachments, respectively. The percentage contribution of area G + H was dramatically greater than that of area E + F at each angle of knee flexion. The anterior fan-like attachment area contributed very little (2% to 3%) to resisting tibial anterior drawer at any angle of knee flexion. The posterior fan-like attachment area contributed 15% ± 6% of the resistance to tibial anterior drawer at 0° of knee flexion, falling to 11% ± 6% at 90°.

##### Tibial Internal Rotation

For the intact knee, the torque required to cause 10° of tibial internal rotation was 5.9 ± 2.5 Nm at full extension. The torque to cause 15° of tibial internal rotation was 4.8 ± 3.7 Nm, 4.2 ± 2.6 Nm, and 5.6 ± 2.3 Nm at 30°, 60°, and 90° of knee flexion, respectively. Cutting the ACL fibers did not have a significant overall effect on the internal rotation torque (*P* = .46 at 0° of knee flexion and *P* > .999 at 30° to 90°), but the small effects were significant as a percentage of the ACL (*P* < .0001) at 0° of flexion (*P* = .725 at 30°, *P* = .300 at 60°, and *P* = .051 at 90°).

**Fig 8:**
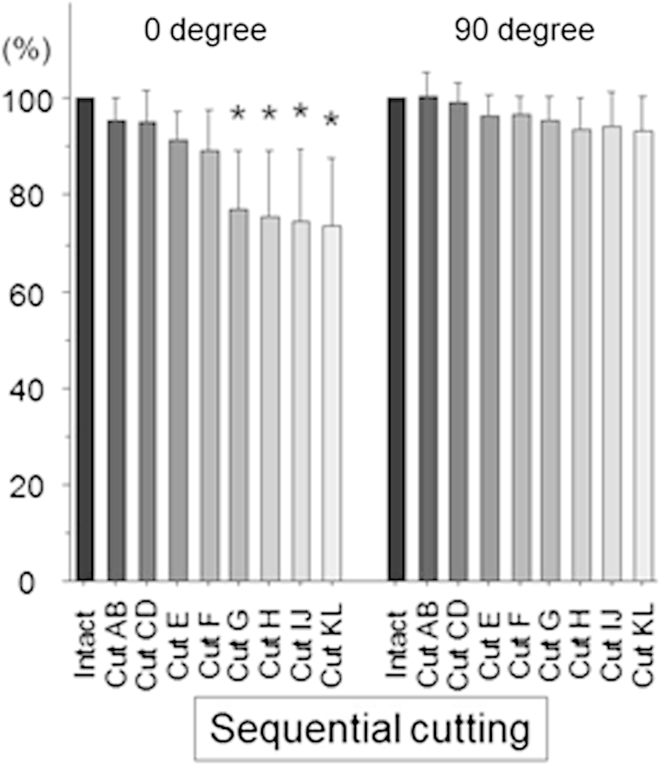
Percentage of torque compared with intact knee in response to 10° of internal rotation at full extension and with 15° of internal rotation at 90° of knee flexion (mean and standard deviation, N = 8). There was a significant decrease after cutting area G at full extension (*P* < .05 [asterisks]).

The percentage of the torque released by each of the sequential cuts was calculated at each angle of knee flexion, when the torque of the intact ACL was considered 100%. There was no significant difference in torque between the intact knee and the partially ACL-deficient knee after cutting areas AB, CD, E, and F at full extension with 10° of internal rotation, although there was a significant decrease in torque after cutting area G (*P* < .0001) (Fig 8). When the ACL had been cut completely, the internal rotation torque had reduced by a mean of 1.7 Nm. The torque required to cause 15° of tibial internal rotation did not reduce significantly with ACL cutting at 30°, 60°, and 90° of knee flexion. The very small changes in tibial internal rotation torque with each stage of cutting the ACL fibers meant that it was not appropriate to attempt to calculate the percentage contributions and resultant forces of each fiber area because they were similar to the precision of the experiment and were too small to have clinical relevance.

##### Tibial External Rotation

In the intact knee, the torque required to cause 10° of tibial external rotation was 3.2 ± 0.8 Nm at full extension. The torque required to cause 15° of tibial external rotation was 2.3 ± 0.5 Nm, 2.8 ± 1.0 Nm, and 3.3 ± 1.2 Nm at 30°, 60°, and 90° of knee flexion, respectively. Cutting the ACL did not affect these torques or the percentage contribution significantly.

## Discussion

The most important findings of this study were that, under the specific experimental conditions, the midsubstance fibers of the ACL (the central attachment areas E, F, G, and H) transmitted 82% to 90% of the resistance to tibial displacement and that the large contribution of the central attachment fibers was biased strongly toward the roof of the femoral intercondylar notch. The fibers attaching to areas G and H, which corresponded to part of the AM bundle, provided from 66% to 84% of the total resistance to anterior drawer across 0° to 90° of flexion (Fig 7). The contribution of fiber attachment areas E and F, which corresponded to part of the PL bundle, fell from 16% at 0° to 9% at 90°. These changes reflected the slackening of the more posterior ACL fibers with knee flexion, which allowed more of the load to fall onto area H, which was “close to isometric.” Similarly, the posterior fan-like extension attachment fibers (areas A, B, C, and D), which form a large part of the attachment area (“footprint”), contributed 15% of the resistance to tibial anterior translation in the extended knee, falling to 11% at 90° (Fig 7). The anterior fan-like extension area contributed only 2% to 3%. Thus this experiment confirmed the hypothesis, formed from anatomic observations, that it was the central direct attachment area that provided most of the resistance to tibial displacement. Furthermore, previous studies that have reported on the centers of the fiber bundle attachments (reviewed by Piefer et al.^23^) have indicated sites more posterior than the center of the dense central attachment area measured in this study, presumably because they included the posterior fan-like fiber attachment area. The implication of this finding is that what are often described as anatomic graft tunnels are more posterior than the main load-bearing zone in the femoral ACL attachment.

**Fig 9:**
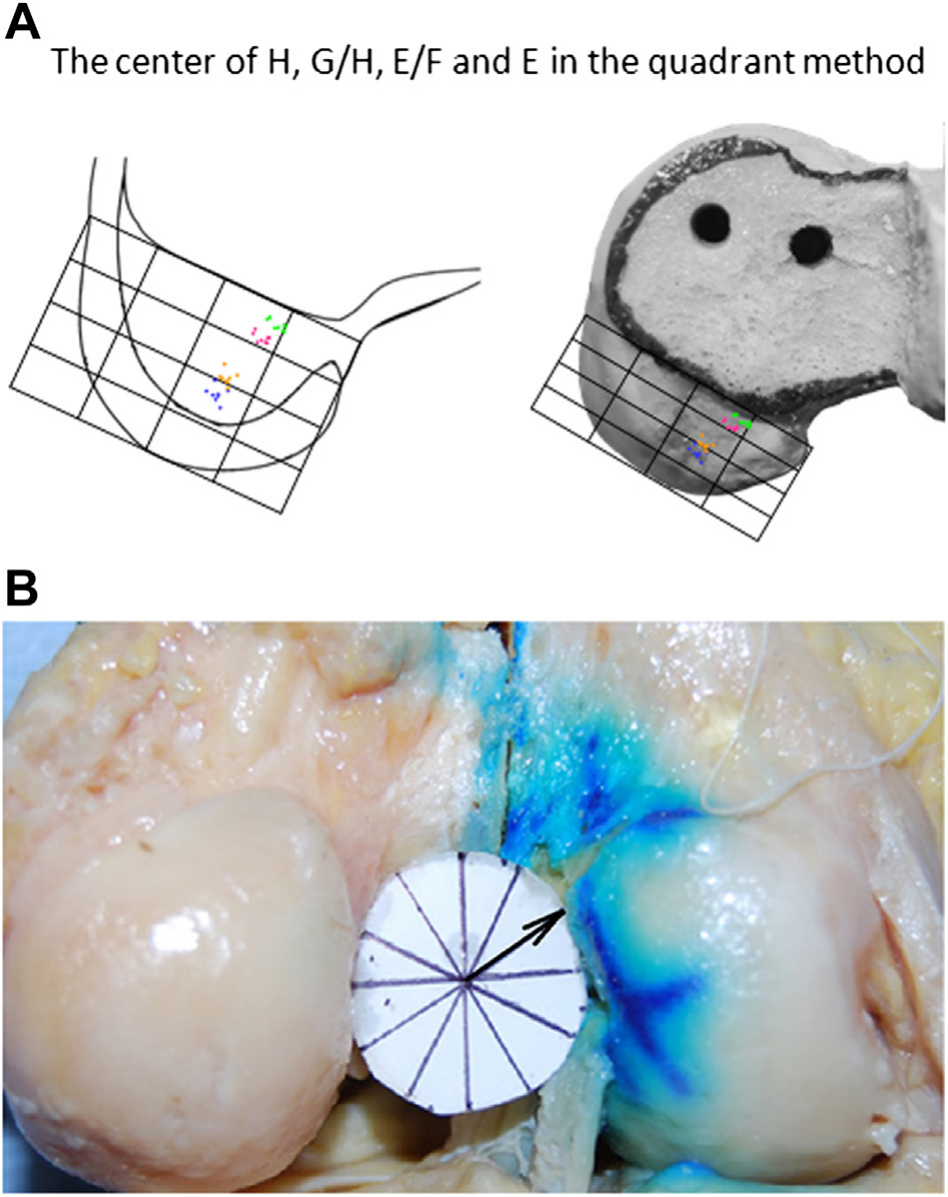
(A) The center of effort of the ACL was, on average, located 30% along the roof of the intercondylar notch and 16% down from the roof, using the grid method of Bernard et al.^25^Table 1 shows the x- and y-coordinates of areas EF, F, GH, and H. (B) The point representing the center of effort of the ACL when viewed through the intercondylar notch from posterior to anterior, parallel to the roof of the notch (Blumensaat's line), was at a mean of 2 hours 2 minutes ± 18 minutes (clock-face position), or 62° ± 5° (mean ± standard deviation). (When the notch was viewed parallel to the long axis of the femur, this angle was at 1 hour 35 minutes ± 13 minutes, or 47° ± 7°.)

This experiment suggests that, in ACL reconstruction, the most important fibers to reproduce the action of the ACL to resist tibial anterior displacement attach to the central/proximal part of the femoral attachment, which approximately corresponds to the AM fiber bundle.^24^ Thus, on the basis of the measurement grid of Bernard et al.,^25^ the graft should be placed at 30% from the posterior margin and 16% down from the roof of the notch to be sited at the “center of effort” of the ACL (Fig 9, Table 1). As the knee flexed, the contributions of the posterodistal parts of the ACL were reduced, concentrating the load further onto the anteroproximal area. This behavior is in line with ACL isometry and fiber length change patterns.^20,26,27^

This study measured the contributions of small fiber bundles located at the ACL femoral attachment to resisting known tibial displacements.^28^ The relative contributions of the ACL fibers may differ if other displacements are used. Those in this study were chosen so as not to induce irreversible changes during repetitive loading. The fibers were resected at the femoral attachment, so we cannot compare the results with the in situ forces after cutting the fiber bundles at the midsubstance^29^ because the orientation of the ACL fibers changes in a more complex pattern at the attachment site than at the midsubstance during knee motion.^16^ Our study shows how fibers attaching to specific areas on the femur resisted tibial displacements. In situ fiber bundle tension has been described,^29^ but it has not been shown how well it acts to stabilize the knee (e.g., if the fiber is vertical). The method used in this work is an updated version of the classic work by Butler et al.,^28^ in which they defined the primary and secondary restraints.

The mechanical function of the ACL found in this study may be related to previous knowledge of its behavior. The fibers that were dominant when resisting tibial anterior translation, near the roof of the femoral intercondylar notch, are tight throughout knee flexion-extension^20,26,27,30^ (i.e., close to “isometric”), and the posterodistal fibers slacken when the knee flexes. These length changes match measurements of how the contribution of the PL bundle falls rapidly with knee flexion.^20^ The mechanical findings of this study match observations of the higher density of collagen fibers in the more anterior part of the cross section of the ACL,^31,32^ which matches the variation of tensile material properties^33^ and of the microscopic morphology of direct fiber insertions into the bone in the central band of the femoral attachment.^12-16^ Conversely, the data raise the question of the role of the fan-like extension areas, given that they appear to carry very little load yet occupy a considerable portion of the attachment area.

Regarding clinical relevance, the results suggest that it may be of less value to create a femoral tunnel in the attachment area of the fan-like extension fibers. This study did not support the concept of trying to cover the entire ACL attachment area with the graft.^8^ Second, in relation to anterior laxity, the results imply that the femoral tunnel of a single-bundle ACL reconstruction in the combined areas G and H would most closely mimic the natural restraint. The data do not support the use of a central “anatomic single-bundle” reconstruction.^8,9^ Concerning double-bundle ACL reconstruction, this study implied that 2 femoral tunnels should be created in the combined areas G + H and combined areas E + F, where the ACL attachment is densest. We believe that this cutting study of the ACL at the femoral attachment increases understanding of the complex functions of the ACL: it provides evidence for when one is considering appropriate tunnel positions on the femur in ACL reconstruction if it is desired that the ACL graft should reproduce the tibial restraining function of the natural ACL.

### Limitations

This study suffered from the limitations of using cadaveric specimens, including that the ACL is much weaker in knees at the age of the specimens used than in younger patients with sports injuries.^34,35^ It was only possible to split the ACL into the 12 fiber areas of the femoral attachment while in situ because of the time taken to run the tests, before degenerative changes occurred in the tissues. The results of cutting at the femoral attachment may differ from those of excision of the whole length of those fibers, down to the tibial plateau. A different cutting sequence may alter the results if load transfer occurs across the width of the ligament. In practice, it was considered too difficult to vary the cutting order among the tiny ligament fiber attachment areas, which would have introduced its own artifacts. Had there been much load transfer, there would have been a large load left acting on the anterior fan-like area, after cutting the central area, and this was not found. Figure 7 shows such an overwhelming majority of the load resistance at area GH that the conclusion of this work would not change even in the presence of significant load transfer across the width of the ACL. Finally, in retrospect, larger amplitudes of tibial internal-external rotation may have led to clearer results regarding the role of the ACL fibers in resisting those rotations. Similarly, greater anterior translation might have shown a clearer role for the fibers of the PL bundle. However, the experimental displacements were chosen such that they would not cause stretching out during the many test repetitions on the partly transected ACL. The work that identified the ACL as being the primary restraint used a 5-mm test displacement.^28^

## Conclusions

Under the experimental conditions described, 66% to 84% of the resistance to tibial anterior drawer force arose from the ACL fibers at the central-proximal area of the femoral attachment close to the roof of the intercondylar notch, corresponding to the AM bundle of the ACL; the fan-like extension fibers contributed very little. This work did not support moving a single-bundle ACL graft down to the side wall of the intercondylar notch or attempting to cover the whole attachment area if the intention was to mimic how the natural ACL resists tibial displacements.

## Figures and Tables

**Table 1:** Locations of ACL Fiber Attachment Areas According to the Radiographic Grid Method of Bernard et al.^25^

ACL Attachment Zone	Shallow-Deep Position, %	High-Low Position, %
E	36.5 (2.3)	55.3 (3.8)
EF	35.1 (2.0)	47.4 (3.3)
F	33.8 (1.8)	39.6 (2.8)
FG	32.4 (1.8)	31.7 (2.4)
G	31.0 (2.0)	23.9 (2.0)
GH	29.6 (2.3)	16.0 (1.7)
H	28.2 (2.8)	8.2 (1.6)

NOTE. Data are presented as mean (standard deviation) of the centers of each of the central direct fiber attachment zones (E, F, G, and H) as a percent of the deep-shallow direction of the grid parallel to the roof of the notch and as a percent of the high-low direction of the grid perpendicular to the roof of the notch. EF, FG, and GH indicate the center of the combined areas of E + F, F + G, and G + H, respectively. These attachment points are shown in Figure 9A.
